# The importance of visual inspection in national quality assurance systems for medicines

**DOI:** 10.1186/s40545-020-00264-w

**Published:** 2020-09-07

**Authors:** Gamal Khalafalla Mohamed Ali, Raffaella Ravinetto, Abubakr Abdelraouf Alfadl

**Affiliations:** 1 Pamela Steele Associates Ltd., Oxford, UK; 2 Department of Public Health, Institute of Tropical Medicine, Nationalestraat 155, 2000 Antwerp, Belgium; 3 Pharmacy Practice Department, Unaizah College of Pharmacy, Qassim University, Buraydah, Saudi Arabia

## Dear Editor,

In their short report, Schiavetti and colleagues presented a visual inspection checklist, designed to guide health workers at the point of care to rapidly identify suspect poor-quality medicines [1]. We would like to emphasize the importance of this tool, by discussing three cases reported at different time points in Sudan.

Since 1998, a simple checklist was implemented by the Revolving Drug Fund (RDF) in Khartoum State, Sudan, and later by the Sudanese National Medical Supplies Fund (NMSF), to monitor the quality of medicines at health facilities in Khartoum State and in Sudan respectively. In 1999, the national lay press widely publicized an incident of contaminated intravenous fluids purchased from an Indian manufacturer [2] and imported by the central medical public corporation (CMS), i.e., the national public procurement agency, which was further transformed into the non-profit NMSF in 2015 [3]. A fungal growth was clearly visible on different bottles from different batches, but those in charge of acting upon the non-conform product disagreed on actions to be undertaken. The “proponents,” mainly at the CMS, argued that findings of the visual inspection should not be generalized to other bottles or other batches that did not show (yet) fungal growth [2], while the “opponents,” mainly at the Chamber of Medicine’s Importers, argued that information from the lay press must be taken seriously, and that batch recalls were urgently needed, as not acting would cause health damage and perhaps claim lives [2]. Eventually, all intravenous fluids imported by CMS from this company were withdrawn from the market, regardless of contamination status, by a ministerial decree in 2001. Remarkably, findings from a simple visual inspection allowed to identify a non-reliable supplier and to prevent future harm.

A similar scenario was repeated in 2005 [3]. At that time, the General Directorate of Pharmacy (G-DOP) was acting as a secretariat of the National Medicines and Poisons Board (NMPB) that is the national medicine regulatory authority (an autonomous secretariat of NMPB, under direct supervision of the Minister of Health, was formally established in November 2007). The G-DOP revoked the marketing authorization of a cough syrup manufactured by a United Arab Emirates-based drug company, and imported by the CMS. The decision was taken because the visual inspection revealed leaked bottles from different batches. Again, decision-makers divided. “Proponents”complained that the G-DOP had not tested the product chemically and based its decision on visual inspection only. The G-DOP argued that when a defect that puts the integrity of a product at stake can be detected visually, there is no need to further test that product chemically.

A third case took place in 2009. According to the Medicines and Poisons Act 2009, the CMS must purchase and supply registered medicines only. Nonetheless, the Act was not fully implemented until 2011. Meanwhile, the CMS imported an unregistered Salbutamol inhaler from China [3], and as a common practice at that time, it sent samples of this unregistered product to NMPB for testing. The NMPB’s National Medicines Quality Control Laboratory (NMQCL) rejected the samples because the country of origin was not stated on both primary and secondary packaging. The CMS insisted that NMQCL should test the product chemically, while the Secretary General of NMPB decided not to do so, based on the definition of counterfeit medicines, as essential information to identify the medicine was missing on the packaging [4]. The discussion scaled up to the Federal Minister of Health, who directed the NMPB secretary general to do the chemical tests for Salbutamol inhaler of unknown source. The tests found that the product contained less than 70% of the stated active ingredient, while it should be at least 90%. In other words, the visual inspection had predicted chemical poor quality, and expensive confirmatory lab test could have been avoided by a less formalistic interpretation of the legislation [5]. In addition, even if the product had passed the chemical tests, an important problem of traceability would have remained.

The checklist of Schiavetti [1] requires to check the name of manufacturer and country of origin. These experiences from Sudan confirm that the availability and reliability of this information is crucial for acceptance of any pharmaceutical products. The level of risk for these parameters should be high (in case of Schiavetti’s checklist, “C” instead of “B” [1]), as lack of information on the origin of a medicine will make traceability impossible and may qualify it as a falsified product [6].

In summary, these real-life cases indicate that a careful visual inspection, i.e., a simple and inexpensive technology, is of paramount importance in monitoring the quality of medicines not only in the field, but also at central level. In a pharmaceutical scenario characterized by a situation of multiple quality standards [7–10], complex distribution networks [11, 12], and weaknesses of the pharmaceutical systems [13, 14], it can provide additional important guidance to timely recall suspicious batches, to revoke marketing authorizations of unreliable suppliers, and to protect public health [15]. Central medical stores and regulatory agencies need to consider the visual inspection as part of their prequalification and ongoing requalification system.

## Data Availability

Not applicable (this letter is based on publicly available information)
